# Case Report: Refractory childhood-onset takayasu arteritis with digital clubbing and extensive supra-aortic and lower-limb arterial disease

**DOI:** 10.3389/fped.2026.1961784

**Published:** 2026-10-20

**Authors:** Fawzy M. Abunejma, Leen M. Jabari, Marah Nouredeen Hamad, Tamer O. Tamimi, Omair Bseiso, Hisham Aziz Dweik, Raed M. A. Isayed, Hanan Qunibi, Waleed Alzaro, Wasef Alhroub

**Affiliations:** 1 Faculty of Medicine, Scientific Research and Medical Development Center, Hebron University, PRCS Hospital, Hebron, Palestine; 2 Hebron Government Hospital, Hebron, Palestine; 3 Palestinian Red Crescent Hospital (PRCSH), Hebron, Palestine; 4 Faculty of Medicine, Palestine Polytechnic University, Al-Ahli Hospital, Hebron, Palestine

**Keywords:** biologic therapy, childhood-onset takayasu arteritis, digital clubbing, large-vessel vasculitis, pediatric rheumatology, tocilizumab, vascular imaging

## Abstract

Childhood-onset Takayasu arteritis is rare, and its early picture is often too vague to point at a large-vessel vasculitis. We report a 12-year-old girl who presented with 13 days of recurrent severe retrosternal chest pain and diffuse colicky abdominal pain, on a background of six months of exertional fatigue and painful swelling of the right fingertips, without fever, weight loss or night sweats. Examination showed bilateral digital clubbing, a right arm blood pressure below the 5th percentile with left arm and lower limb readings between the 50th and 90th percentiles, an absent right radial pulse, and weak brachial and right posterior tibial pulses. The erythrocyte sedimentation rate was 110 mm/h and C-reactive protein 82 to 93 mg/L. Computed tomography angiography showed mild concentric mural thickening of the aortic arch with severe stenosis of the right common and internal carotid arteries, near-occlusion of the right subclavian artery, moderate to severe brachiocephalic stenosis, moderate narrowing of the left common carotid and left subclavian arteries, and moderate stenosis of the right common and superficial femoral arteries. The abdominal aorta and its visceral branches were spared. She met the EULAR/PRINTO/PRES (Ankara 2008) classification criteria for childhood Takayasu arteritis, and the arterial distribution corresponds to Numano type IIa, together with peripheral lower-limb involvement that the Numano scheme does not cover. Disease progressed on intravenous methylprednisolone, oral prednisolone, methotrexate, aspirin and low-molecular-weight heparin, culminating in an 80% stenosis of the right common femoral artery. Adalimumab was started and later replaced by tocilizumab, after which the femoral peri-arterial inflammatory changes resolved on imaging and acute-phase reactants normalized. Blood pressure asymmetry, pulse deficits and the established stenoses persisted. This case suggests that normal inflammatory markers in childhood-onset Takayasu arteritis can coexist with fixed arterial damage, and that judgements about disease activity are better based on clinical findings and serial imaging together than on acute-phase reactants alone.

## Introduction

Takayasu arteritis (TAK) is the most common large-vessel vasculitis of childhood, although it remains rare in absolute terms. It is characterized by chronic, autoimmune, granulomatous inflammation of the aorta and its major branches, which leads to mural thickening, stenosis and thrombus formation; aneurysms and dissection are also seen (1, 2). The disease is predominantly described in women under 40 years of age (2). About 30% of patients with TAK develop symptoms before adulthood, a group defined as childhood-onset Takayasu arteritis (cTAK), and most pediatric cases are diagnosed between 10 and 15 years of age (3, 4).

Because cTAK is rare and its manifestations are heterogeneous, recognition is often late. Early symptoms tend to be nonspecific and systemic, with fatigue, fever and weight loss, so the initial phase can pass unrecognized. Hypertension, diminished or asymmetric pulses and features of end-organ ischemia appear as disease progresses and usually raise the suspicion of large-vessel vasculitis. Without timely treatment, cTAK carries a high disease burden, frequent flares and early vascular damage (2).

Vascular imaging is central to both diagnosis and monitoring, since angiographic studies define the extent of arterial involvement and can distinguish inflammatory change from established structural damage. Assessing activity nonetheless remains difficult. Erythrocyte sedimentation rate (ESR) and C-reactive protein (CRP) may not track arterial wall inflammation, and current recommendations therefore integrate clinical, laboratory and imaging findings rather than relying on any single measure (5, 6).

Management of childhood-onset disease is largely extrapolated from adult studies. High-dose glucocorticoids remain the cornerstone of induction, but relapse rates are high and prolonged exposure is particularly costly in children, so a non-glucocorticoid immunosuppressive agent such as methotrexate or azathioprine is usually added early (7, 8, 20). Tumor necrosis factor (TNF) inhibitors and interleukin-6 (IL-6) receptor antagonists have shown promise in refractory disease, although comparative pediatric data are scarce (8). The absence of a validated biomarker of arterial inflammation compounds the problem (9).

We report a 12-year-old girl with cTAK who presented atypically, had extensive supra-aortic and peripheral arterial disease, and continued to progress on conventional and biologic immunosuppression. The report follows the CARE guidelines (24). It is retrospective, so its scope is limited to what the clinical record documents, and we have indicated throughout where an investigation was not performed or a value was not retrievable.

## Case presentation

### Patient information and presenting complaints

A 12-year-old previously healthy girl presented in January 2026 with a 13-day history of recurrent, severe, heavy retrosternal chest pain and diffuse colicky abdominal pain. Episodes occurred two to three times daily, lasted about 15 min, resolved spontaneously and often woke her from sleep. For six months beforehand she had noticed progressive exertional fatigue together with intermittent pain and “drumstick” swelling of the fingertips of her right hand. She had no fever, weight loss, night sweats or joint pain.

### Clinical findings

She had prominent bilateral digital clubbing and marked four-limb blood pressure asymmetry. Right arm blood pressure was below the 5th percentile for age and sex, while left arm and lower limb readings fell between the 50th and 90th percentiles. The right radial pulse was absent, the left radial pulse was markedly diminished, both brachial pulses were weak, and the right posterior tibial pulse was weak. The combination of pulse deficits, inter-limb blood pressure discrepancy and systemic inflammation in an adolescent girl raised immediate suspicion of large-vessel vasculitis.

### Diagnostic assessment

#### Laboratory findings

Laboratory testing at presentation showed a marked systemic inflammatory response, with an ESR of 110 mm/h and CRP between 82 and 93 mg/L, accompanied by mild normocytic anemia, leukocytosis and thrombocytosis. Serum complement was reported as elevated. Antinuclear antibody, anti-double-stranded DNA, *p*-ANCA, c-ANCA and serum angiotensin-converting enzyme were negative, and anti-Saccharomyces cerevisiae antibody (ASCA) IgA, which was requested because of the prominent abdominal pain and the possibility of inflammatory bowel disease, was also negative. At the later admission described below, the white cell count was mildly elevated, hemoglobin was stable, and ESR and CRP had returned to normal.

We are not able to report numeric complement values with their reference ranges, or a full set of serial laboratory values across the treatment course, because these were not retrievable from the record available for this report. A table of serial laboratory data would have strengthened the report, and we acknowledge its absence as a limitation.

#### Imaging

Doppler ultrasonography and computed tomography angiography (CTA) of the aorta and its branches were performed at diagnosis. The aortic arch showed mild concentric mural thickening without stenosis, aneurysmal change or thrombus. The supra-aortic branches were extensively involved: severe stenosis of the right common and internal carotid arteries, near-complete occlusion of the right subclavian artery, moderate to severe stenosis of the brachiocephalic artery, and moderate narrowing of the left common carotid and left subclavian arteries. Diffuse concentric wall thickening with mural enhancement was present in the affected segments. In the lower limbs there was moderate stenosis of the right common femoral and superficial femoral arteries with concentric wall thickening. The ascending aorta, the descending thoracic aorta, the abdominal aorta, the coeliac, superior mesenteric and inferior mesenteric arteries, and both renal arteries showed no wall thickening, enhancement, stenosis or occlusion. No aneurysmal dilatation or intraluminal thrombus was identified in any territory at any point. Findings by territory, including every segment verified as normal, are set out in Table 1, and representative images are shown in Figure 1.

**Table 1:** Arterial findings by territory on computed tomography angiography at diagnosis, with documented interval change.1.

Arterial territory	At diagnosis	Documented interval change
Ascending aorta	No abnormality	*Not re-imaged*
Aortic arch	Mild concentric mural thickening; no stenosis, aneurysm or thrombus	*Not systematically re-imaged*
Brachiocephalic artery	Moderate to severe stenosis with concentric wall thickening	Mild luminal improvement on early follow-up Doppler
Right common carotid	Severe stenosis; concentric wall thickening with mural enhancement	Mild luminal improvement on early follow-up Doppler
Right internal carotid	Severe stenosis	*Not systematically re-imaged*
Left common carotid	Moderate stenosis	*Not systematically re-imaged*
Right subclavian	Near-complete occlusion	Mild luminal improvement on early follow-up Doppler
Left subclavian	Moderate stenosis	Mild luminal improvement on early follow-up Doppler
Descending thoracic aorta	No abnormality	*Not re-imaged*
Abdominal aorta	Spared	*Not re-imaged*
Coeliac, superior and inferior mesenteric arteries	Patent, normal calibre and wall thickness	*Not re-imaged*
Renal arteries	Spared; preserved renal perfusion on Doppler	Preserved renal perfusion on repeat Doppler
Right common femoral	Moderate stenosis with concentric wall thickening	Progressed to 80% stenosis with diffuse mural thickening and peri-arterial fat stranding; inflammatory change resolved after tocilizumab, with residual wall thickening
Right superficial femoral	Moderate stenosis with concentric wall thickening	Persistent wall thickening in the lower limb arteries after tocilizumab
Coronary arteries	*Not imaged*	*Not imaged*
Pulmonary arteries	*Not specifically imaged*	*Not specifically imaged*

No aneurysmal dilatation or intraluminal thrombus was identified in any territory at any point. Systematic per-territory follow-up imaging is not available, and italicized entries indicate this rather than a normal result. The normal descending thoracic aorta excludes the type IIb component of the Numano classification, and the spared abdominal aorta and renal arteries exclude the type IV component, so the distribution is not type V. The right common and superficial femoral stenoses lie outside the segments the Numano scheme covers and are reported separately as peripheral involvement.

**Figure 1:**
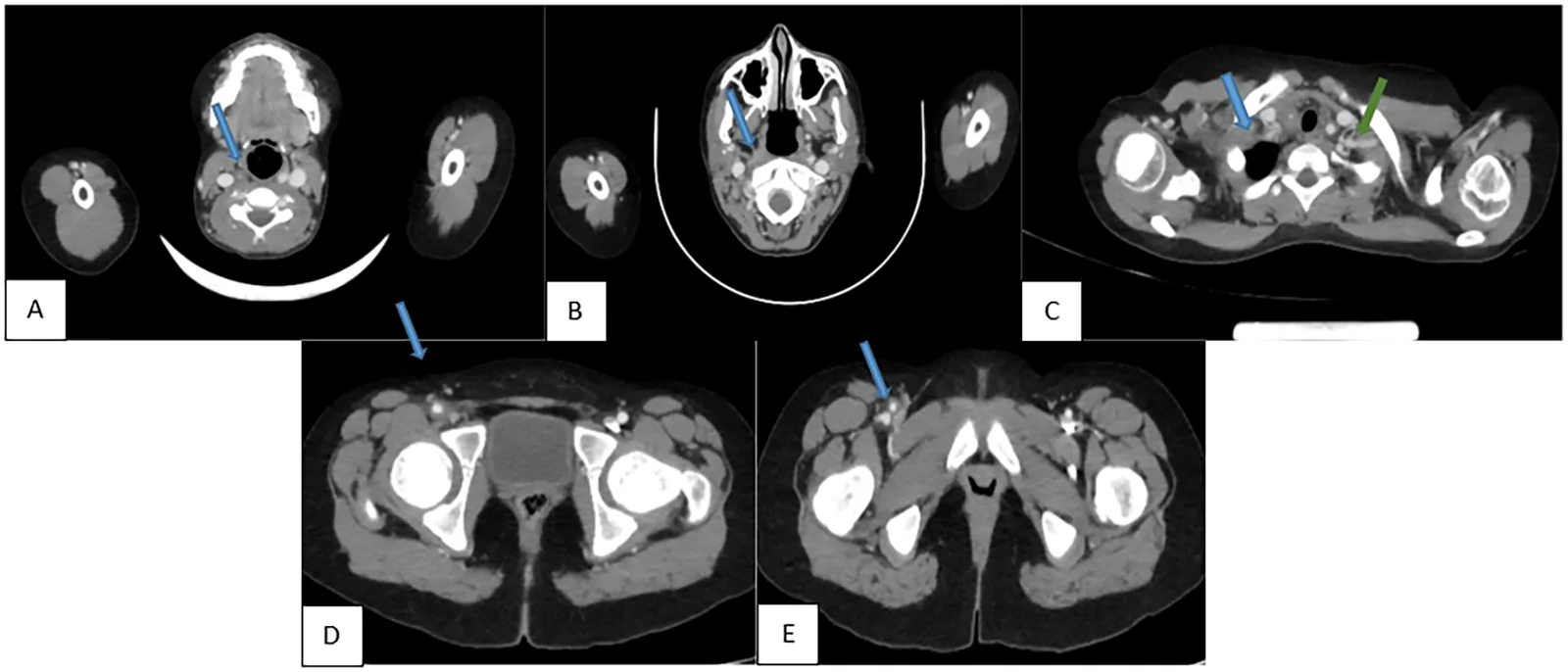
Selected computed tomography angiography images at diagnosis. Panels **A** to **E** show the right-sided arterial lesions described in the text: severe stenosis of the right common and internal carotid arteries, near-complete occlusion of the right subclavian artery, and stenosis of the right common and superficial femoral arteries. Arrows indicate the site of maximal luminal narrowing.

#### Investigations not performed or not available

Both reviewers of this report reasonably asked what else was done, and we prefer to set out the gaps rather than leave them to be inferred. The coronary and pulmonary arteries were not specifically imaged, so the C and *P* modifiers of the Numano classification cannot be applied. Magnetic resonance angiography and 18F-FDG PET-CT were not available at our centre and were not performed; Doppler ultrasonography was used for interval assessment because it is repeatable and avoids further radiation exposure in a child. Resting and exertional oxygen saturation, electrocardiography, cardiac biomarkers, pulmonary function testing, a formal shunt study, a thrombophilia screen, ophthalmologic examination and genetic testing for the monogenic vasculopathies are not documented in the record. No validated disease-activity instrument such as ITAS2010 (21) was applied prospectively. Each of these is carried into the limitations paragraph below.

#### Classification and fulfilment of diagnostic criteria

The diagnosis rested on angiographic abnormality of the aorta and its main branches together with supporting clinical and laboratory features, and the patient fulfilled the EULAR/PRINTO/PRES (Ankara 2008) classification criteria for childhood Takayasu arteritis (18). The mandatory angiographic criterion was satisfied by the CTA findings above, and four of the five additional criteria were met (Table 2). Fibromuscular dysplasia was considered and judged unlikely given the concentric circumferential mural thickening with enhancement, the supra-aortic distribution, the absence of the string-of-beads appearance in the renal arteries, and the accompanying acute-phase response. The 2022 ACR/EULAR classification criteria for Takayasu arteritis were also met, although these were developed and validated in adults (14, 15).

**Table 2:** Fulfilment of the EULAR/PRINTO/PRES (Ankara 2008) classification criteria for childhood takayasu arteritis (18).

Criterion	Requirement	Findings in this patient
Mandatory	Angiographic abnormality of the aorta or its main branches, or the pulmonary arteries, showing aneurysm, dilatation, narrowing, occlusion or thickened arterial wall, not due to fibromuscular dysplasia	Met. CTA showed mild concentric mural thickening of the aortic arch with severe stenosis of the right common and internal carotid arteries, near-occlusion of the right subclavian artery, moderate to severe brachiocephalic stenosis, and moderate left common carotid and left subclavian stenosis. Concentric circumferential thickening with mural enhancement and the supra-aortic distribution argue against fibromuscular dysplasia
1. Pulse deficit or claudication	Lost, weakened or absent peripheral artery pulse, or claudication	Met. Absent right radial pulse, markedly diminished left radial pulse, weak bilateral brachial and weak right posterior tibial pulses; right hip pain and limp with exertion
2. Blood pressure discrepancy	Difference of more than 10 mmHg in systolic blood pressure between any limbs	Met. Right arm below the 5th percentile with left arm and lower limb readings between the 50th and 90th percentiles
3. Bruits	Audible murmurs or palpable thrills over large arteries	*Not documented; auscultation over the large arteries is not recorded in the clinical notes*
4. Hypertension	Systolic or diastolic blood pressure above the 95th percentile for height	Met at the later admission (mild hypertension); not present at first presentation
5. Acute-phase reactants	ESR above 20 mm/h or CRP above the normal range	Met. ESR 110 mm/h; CRP 82 to 93 mg/L

CRP, C-reactive protein; CTA, computed tomography angiography; ESR, erythrocyte sedimentation rate. The criteria require the mandatory angiographic criterion plus at least one of the five additional criteria; four of the five were met here.

Applying the Numano angiographic classification to the territories actually involved, the pattern corresponds to type IIa, that is, the ascending aorta, aortic arch and its branches (19). Neither component required for a type V designation was present: the descending thoracic aorta showed no abnormality, which excludes type IIb, and the abdominal aorta and renal arteries were spared, which excludes type IV. The stenoses of the right common femoral and superficial femoral arteries lie outside the segments the Numano scheme describes, so they cannot be used to upgrade the classification and are reported here as separate peripheral involvement. We consider this distribution, supra-aortic disease of this severity in combination with symptomatic femoral stenosis, to be the more clinically informative description in a child of this age.

#### Differential diagnosis

Several alternatives were considered in a 12-year-old with chest pain, abdominal pain, digital clubbing, pulse and blood pressure asymmetry, systemic inflammation and multifocal arterial stenoses. Infective endocarditis and mycotic aneurysm were considered because of the inflammatory response and the peripheral vascular findings; echocardiography showed no vegetation and there were no embolic phenomena or cutaneous stigmata of endocarditis. Tuberculous aortitis is a relevant consideration in our region, and there was no pulmonary or nodal disease on cross-sectional imaging, although a systematic infectious work-up is not documented. Systemic lupus erythematosus, antiphospholipid syndrome and other connective tissue diseases were considered on the basis of the multisystem presentation, and the serological panel was negative. ANCA-associated vasculitis is not typically a large-vessel disease, and both *p*-ANCA and c-ANCA were negative. Sarcoidosis was considered because of the elevated acute-phase reactants and was not supported by a negative angiotensin-converting enzyme level or by the imaging. Inflammatory bowel disease with extraintestinal vasculitis was raised by the abdominal pain and prompted ASCA testing, which was negative, and there was no diarrhoea or rectal bleeding. Fibromuscular dysplasia, mid-aortic syndrome and the monogenic vasculopathies and vasculitis mimics of childhood, including neurofibromatosis type 1, Williams syndrome, deficiency of adenosine deaminase 2 and the heritable connective tissue disorders, were considered against the clinical phenotype, which showed none of the dysmorphic, cutaneous, neurologic or hematologic features that characterize them; genetic testing was not available at our centre. We would emphasize that negative serology reduces the likelihood of these conditions rather than excluding them, and that the diagnosis of Takayasu arteritis here rests on the imaging distribution together with the clinical criteria, not on the negative panel.

#### The digital clubbing

Clubbing involved the fingers of both hands and had begun in the right fingertips six months before presentation. It is one of the more unusual features of this case, and we want to be careful about what can be concluded from it. Clubbing in a child is more often cardiac, pulmonary, hepatic, gastrointestinal or endocrine in origin than vascular, and those causes were not systematically investigated here. There was no cyanosis. Echocardiography reported only mild tricuspid regurgitation and mildly reduced left ventricular systolic function, with no intracardiac shunt and no structural congenital heart disease described. Gastrointestinal symptoms were limited to the colicky pain above, with a negative ASCA IgA. Beyond that, pulmonary function testing, dedicated pulmonary artery imaging, a formal shunt study and a hepatic work-up were not performed, so an occult pulmonary, cardiac or hepatic contribution cannot be excluded, and we say so rather than implying that the arteritis caused the clubbing. Chronic arterial insufficiency of the upper limbs, which is documented here, has been reported alongside clubbing in Takayasu arteritis, although only a small number of such cases exist and the mechanism proposed for them, local hypoxia with release of platelet-derived and vascular endothelial growth factors, remains hypothetical (23). We regard the association in our patient as unexplained rather than causal.

#### The chest and abdominal pain

The mechanism of the presenting pain was not established with certainty and deserves explicit comment, since the abdominal aorta and visceral branches were spared. Mesenteric ischemia was considered and was not supported: the coeliac, superior mesenteric and inferior mesenteric arteries were specifically assessed on CTA and were patent, of normal calibre and without wall thickening, the pain was not consistently postprandial, and there was no diarrhoea, gastrointestinal bleeding or weight loss. Renal Doppler ultrasonography showed preserved renal perfusion. No structural abdominal cause was found, and the pain settled as systemic inflammation came under control, so a nonspecific inflammatory or referred mechanism seems the most likely explanation. Small-vessel mesenteric involvement below the resolution of CTA cannot be excluded.

For the chest pain, the aortic root and ascending aorta were normal on CTA and there was no aortic regurgitation. Electrocardiography and cardiac biomarkers are not documented, and the coronary arteries were not imaged. When she was readmitted with recurrent severe nocturnal right-sided chest pain, she was mildly hypertensive, echocardiography showed mild tricuspid regurgitation with mildly reduced left ventricular systolic function, and acute-phase reactants were normal. Taking the supra-aortic disease, the reduced ventricular function and the mild hypertension together, we consider the chest pain most likely multifactorial, with hypertensive and demand-related mechanisms contributing. Coronary involvement was not formally excluded and remains a possibility.

### Therapeutic intervention

Management was multidisciplinary from the outset, involving pediatric rheumatology, vascular surgery, cardiology, infectious disease, hematology and radiology. Induction consisted of pulsed intravenous methylprednisolone 20 mg/kg followed by oral prednisolone 1 mg/kg/day. Weekly subcutaneous methotrexate 15 mg/m^2^ was added as the non-glucocorticoid immunosuppressive agent, in line with guidance that recommends a glucocorticoid combined with a second agent for active large-vessel vasculitis (20). The sequence of agents, the indication for each and the reason for each change are set out in Table 3. Exact doses, dosing intervals and treatment durations for adalimumab, tocilizumab, aspirin, low-molecular-weight heparin and enalapril, and the schedule of the corticosteroid taper, were not recorded in the material available for this report. We present the sequence and the reasoning rather than dosing we cannot verify, and we note the omission in the limitations.

**Table 3:** Treatment sequence, with the indication for each agent and the reason for each change.

Stage	Agent	Dose and route	Indication or reason for change
Induction	Methylprednisolone	20 mg/kg/day intravenous pulse	Active large-vessel vasculitis with critical supra-aortic stenosis
Induction	Prednisolone	1 mg/kg/day orally, subsequently tapered	Maintenance of induction; taper schedule not recorded
Induction	Methotrexate	15 mg/m^2^ subcutaneously once weekly	Non-glucocorticoid immunosuppressive agent added per guideline recommendation (20)
Induction	Aspirin	*Low dose, orally; exact dose not recorded*	Antiplatelet cover for critical stenosis
Acute femoral event	Low-molecular-weight heparin	*Therapeutic dose; agent and dose not recorded*	Empirical, for near-occlusive subclavian disease with an acute high-grade femoral stenosis. No documented thrombus
First escalation	Adalimumab	*Not recorded*	Progression on glucocorticoid and methotrexate: persistent active arteritis on CTA and progression of the right common femoral lesion to 80% stenosis with hip pain and limp
Second escalation	Tocilizumab	*Intravenous; dose and interval not recorded*	Continued vascular progression on adalimumab. IL-6 receptor blockade selected on pathogenetic grounds, supporting pediatric switching data (8, 17), and local availability
Later admission	Methylprednisolone	*Intravenous pulse; dose not recorded*	Recurrent chest pain at readmission
Later admission	Enalapril	*Orally; dose not recorded*	Mild left ventricular systolic dysfunction and hypertension

CTA, computed tomography angiography; IL-6, interleukin-6. Italicized entries indicate data not retrievable from the record available for this report, which is acknowledged in the limitations. Methotrexate was continued throughout with folic acid supplementation.

Antiplatelet therapy with low-dose aspirin was given for critical supra-aortic and, later, femoral stenosis, with the aim of reducing the risk of thrombotic occlusion of an already narrowed lumen. Therapeutic low-molecular-weight heparin was started empirically at the time of the acute femoral event rather than as treatment of a documented thrombus. No intraluminal thrombus was identified on CTA at any point, and no thrombophilia screen was performed. The indication was therefore the combination of near-occlusive subclavian disease and an acute high-grade femoral stenosis with limb symptoms, and the decision was taken jointly with hematology and vascular surgery after weighing the bleeding risk in a 12-year-old. No bleeding complication occurred. In retrospect we regard this as a pragmatic decision taken under uncertainty rather than an evidence-based one, and we would not present it as standard practice.

Early follow-up Doppler imaging showed mild luminal improvement in the upper limb branches. A repeat CTA, however, showed persistent active arteritis with diffuse mural thickening and peri-arterial fat stranding, together with progression of the right common femoral artery lesion. She then developed severe right hip pain and a limp from an 80% stenosis of the right common femoral artery. This was treated medically; revascularization was discussed with vascular surgery and deferred because collateral supply was adequate, there was no critical limb ischemia, and active inflammation makes early intervention prone to restenosis. Because progression had occurred on adequate doses of glucocorticoid and methotrexate, therapy was escalated to the TNF inhibitor adalimumab.

Vascular disease continued to progress on adalimumab, and treatment was therefore switched to the IL-6 receptor antagonist tocilizumab. Tocilizumab was chosen over a second TNF inhibitor or a conventional agent because IL-6 has a well-described role in the pathogenesis of Takayasu arteritis, because pediatric series report benefit when switching between anti-TNF and anti-IL-6 agents in refractory disease (8, 17), and because it was the biologic accessible to us at the time. Following the switch, imaging showed resolution of the acute femoral peri-arterial inflammatory change, with persistent arterial wall thickening in the lower limbs. At her most recent admission she received a further pulse of intravenous methylprednisolone, methotrexate was continued, the next scheduled dose of intravenous tocilizumab was given, and enalapril was started for mild left ventricular dysfunction and hypertension. Treatment was tolerated without infusion reaction, cytopenia, transaminitis or serious infection over the period reported. The sequence of clinical events, major imaging findings, and therapeutic interventions from presentation in January 2026 through the most recent admission in June 2026 is summarized in Figure 2.

**Figure 2:**
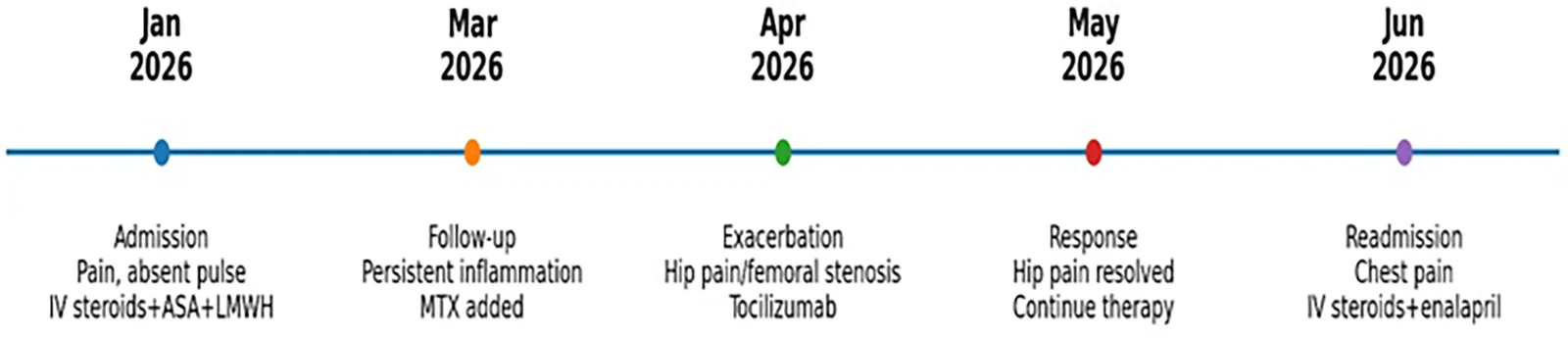
Clinical timeline from presentation in January 2026 to the most recent admission in June 2026, showing the presenting and interval symptoms, the main imaging findings, and each treatment started or changed. The reasoning behind each treatment change, and the indication for each agent, are set out in Table 3.

### Follow-up and outcomes

She achieved clinical stability without acute ischemic complication and was discharged in ambulatory condition. By then the hip pain and limp had resolved, ESR and CRP were normal, and hemoglobin was stable. Her examination, however, was unchanged in the respects that matter: four-limb blood pressure remained asymmetric, with right arm readings persistently below the 5th percentile, the right radial pulse remained absent, and the left radial and brachial pulses remained diminished. Renal Doppler confirmed preserved renal perfusion.

Documented follow-up ends at the admission of June 2026, giving an observation period of approximately five months from presentation and a considerably shorter interval since tocilizumab was started. We state this plainly because it is a real constraint on the report. Structured follow-up data beyond that admission, including repeat imaging of every territory, later laboratory values and later echocardiography, are not available, and nothing here should be read as evidence of sustained remission. She remains under joint rheumatology, cardiology and vascular surgery surveillance, with serial Doppler ultrasonography and periodic CTA planned to detect progression and to time any future revascularization.

### Patient and parental perspective

The CARE guidelines recommend including the patient perspective where possible (24). A structured perspective statement was not recorded during the episode of care and could not be obtained retrospectively for this report, and we would rather say so than offer a paraphrase we cannot attribute to the family. Written informed consent for publication of the clinical details and images was provided by the patient's parents and legal guardian.

## Discussion

Childhood-onset Takayasu arteritis is a chronic granulomatous large-vessel vasculitis that mainly targets the aorta and its main branches (10). Roughly 30% of patients with TAK develop symptoms before adulthood, and pediatric onset clusters in early adolescence, with a mean age at presentation of about 12 years (10, 11). Pathogenesis involves an interplay of genetic susceptibility, innate immune activation and adaptive immune responses. The strongest and most consistently replicated genetic association is with HLA-B*52, which is linked both to susceptibility and to clinical phenotype across populations (22). Inflammatory infiltration of the arterial wall by dendritic cells, T lymphocytes, macrophages and natural killer cells drives the release of TNF-alpha, IL-6, interferon-gamma and IL-17, with destruction of elastic tissue, vascular remodelling, fibrosis and progressive luminal narrowing. Those pathways are the rationale for TNF and IL-6 receptor blockade in refractory disease (12). Three aspects of the present case seem worth drawing out.

### A typical presentation and diagnostic recognition

Our patient had extensive arterial disease and almost none of the constitutional features that classically prompt a vasculitis work-up. Fever, weight loss and night sweats were all absent. What she had instead was pain, fatigue and a set of findings that only appear if somebody examines the pulses and measures the blood pressure in all four limbs. Pediatric cohorts describe the same pattern, with children more often presenting with advanced vascular manifestations, hypertension, abdominal pain or cardiac involvement than with an inflammatory prodrome, and with a correspondingly aggressive vascular phenotype (10, 13). The practical implication is narrow and worth stating: in a child with unexplained systemic inflammation, a four-limb blood pressure measurement and a careful pulse examination cost nothing and, in this case, were what redirected the work-up. Digital clubbing was the other unusual feature. Only a handful of cases of clubbing in Takayasu arteritis have been published, and the proposed mechanism of local hypoxia with growth-factor release remains speculative (23). In our patient, clubbing coexisted with severe upper limb arterial insufficiency, but the pulmonary, cardiac and hepatic causes were not systematically investigated, so we treat the association as an observation rather than as evidence of causation.

### Defining disease activity when markers and imaging disagree

The most instructive part of this case is also the part where terminology has to be handled carefully. ESR and CRP normalized, while the pulse deficits, the blood pressure asymmetry and the established stenoses did not. These are not contradictory observations. Fixed stenosis and occlusion are the structural residue of previous inflammation and can persist indefinitely after inflammation has been suppressed; conversely, interval structural progression may indicate that inflammation is continuing. Distinguishing the two requires more than acute-phase reactants, and in a patient receiving tocilizumab it requires considerably more, since IL-6 receptor blockade suppresses hepatic CRP synthesis and lowers ESR independently of vascular inflammation (9). For that reason we have avoided the term biochemical remission in this report. The treating team defined active disease as new or worsening ischemic symptoms, new or progressive stenosis or new mural thickening and enhancement on imaging, or a rise in acute-phase reactants not attributable to another cause; improvement as the resolution of ischemic symptoms with regression of mural thickening or enhancement; and irreversible damage as stenosis or occlusion persisting on serial imaging without accompanying wall enhancement or peri-arterial change. No validated activity instrument was applied prospectively, which we count as a limitation. Current imaging recommendations for large-vessel vasculitis emphasize exactly this integration of clinical, laboratory and imaging assessment (5, 6).

### Escalation of immunosuppression versus structural recovery

Pediatric treatment decisions in this disease are made on thin evidence. Guidance supports a glucocorticoid with a non-glucocorticoid immunosuppressive agent for active disease and offers only conditional recommendations on TNF inhibitors and tocilizumab, largely extrapolated from adult data (20). Our patient progressed on glucocorticoid and methotrexate, progressed again on adalimumab, and was switched to tocilizumab, an approach supported by pediatric series reporting benefit from switching between anti-TNF and anti-IL-6 agents in refractory cTAK (8, 17) and by comparative adult data (12). After the switch, the peri-arterial fat stranding and mural enhancement around the right common femoral artery resolved on imaging, her ischemic limb symptoms settled and acute-phase reactants normalized. What did not change was the calibre of the affected arteries. It would overstate the case to say that tocilizumab improved vascular inflammation on the strength of symptoms and blood tests alone; the imaging change described above is the specific evidence, and it is limited to one territory over a short interval. The broader point is that suppressing inflammation and restoring vascular anatomy are different endpoints, and only the first is within reach of immunosuppression once stenosis is established. That is an argument for early recognition, and for keeping revascularization under active review in a multidisciplinary setting rather than treating medical therapy as the whole of management (16).

### Strengths and limitations

The strengths of this report are a detailed bedside vascular examination with four-limb blood pressure documentation, longitudinal imaging across two modalities, a treatment course that spans conventional and two classes of biologic therapy, and management by a genuinely multidisciplinary team. The limitations are substantial, and we would rather state them than have them inferred. This is a single patient, so no causal inference is possible and generalizability is limited. Documented follow-up ends at the June 2026 admission, an observation period of about five months and shorter still since tocilizumab began, so nothing here supports a claim of sustained remission. The report is retrospective, and several categories of data are simply absent: numeric complement values with reference ranges and a full set of serial laboratory values; exact doses, intervals and durations for adalimumab, tocilizumab, aspirin, low-molecular-weight heparin and enalapril, together with the corticosteroid taper schedule; electrocardiography and cardiac biomarkers; resting and exertional oxygen saturation; and a structured patient and family perspective. Systematic follow-up imaging of every arterial territory is not available, so interval change can be reported only where it was documented. The investigations that would have addressed pulmonary, cardiac and hepatic causes of clubbing most directly were not performed, and the mechanism of the clubbing therefore remains unexplained. Coronary and pulmonary arteries were not imaged, so the C and *P* modifiers of the Numano classification could not be assigned. Ophthalmologic examination, thrombophilia screening and genetic testing for the monogenic vasculopathies were not performed, and MR angiography and PET-CT were unavailable. No validated disease-activity score was applied prospectively. Acute-phase reactants are imperfect markers of arterial inflammation and are further confounded by IL-6 receptor blockade, which limits how far the laboratory course can be interpreted, and distinguishing ongoing arteritis from fixed vascular damage on CTA alone is not always possible. Finally, the pediatric evidence base for sequential biologic therapy is thin, so our treatment decisions reflect clinical judgement and local availability as much as published data.

## Conclusion

Childhood-onset Takayasu arteritis can present without the constitutional symptoms that usually prompt a vasculitis work-up, and it can progress while a child is on apparently adequate immunosuppression. This case suggests two things. Careful vascular examination, including four-limb blood pressure measurement, can redirect the diagnostic pathway in a child with unexplained inflammation. And normal acute-phase reactants should not be read as evidence that the arteries are quiescent or recovering, particularly during IL-6 receptor blockade, which is an argument for defining activity from clinical findings and serial imaging together. Larger pediatric studies are needed before treatment escalation in refractory disease can rest on anything firmer than extrapolation.

## Data Availability

The raw data supporting the conclusions of this article will be made available by the authors, without undue reservation.
